# High-definition transcranial direct current stimulation for upper extremity rehabilitation in moderate-to-severe ischemic stroke: a pilot study

**DOI:** 10.3389/fnhum.2023.1286238

**Published:** 2023-10-12

**Authors:** Jordan N. Williamson, Shirley A. James, Dorothy He, Sheng Li, Evgeny V. Sidorov, Yuan Yang

**Affiliations:** ^1^Department of Bioengineering, Grainger College of Engineering, University of Illinois Urbana-Champaign, Urbana, IL, United States; ^2^University of Oklahoma Health Sciences Center, Hudson College of Public Health, Oklahoma City, OK, United States; ^3^University of Oklahoma Health Sciences Center, College of Medicine, Oklahoma City, OK, United States; ^4^Department of Physical Medicine and Rehabilitation, UT Health Huston, McGovern Medical School, Houston, TX, United States; ^5^Department of Neurology, University of Oklahoma Health Sciences Center, Oklahoma City, OK, United States; ^6^Clinical Imaging Research Center, Stephenson Family Clinical Research Institute, Carle Foundation Hospital, Urbana, IL, United States; ^7^Beckman Institute for Advanced Science and Technology, University of Illinois Urbana-Champaign, Urbana, IL, United States; ^8^Department of Physical Therapy and Human Movement Sciences, Northwestern University, Chicago, IL, United States; ^9^Department of Rehabilitation Sciences, College of Allied Health, University of Oklahoma Health Sciences Center, Oklahoma City, OK, United States; ^10^Gallogly College of Engineering, Stephenson School of Biomedical Engineering, University of Oklahoma, Oklahoma City, OK, United States

**Keywords:** transcranial direct current stimulation, transcranial magnetic stimulation, stroke, upper extremity rehabilitation, motor evoked potential

## Abstract

**Introduction:**

Previous studies found that post-stroke motor impairments are associated with damage to the lesioned corticospinal tract (CST) and hyperexcitability of the contralesional cortico-reticulospinal tract (CRST). This proof-of-concept study aims to develop a non-invasive brain stimulation protocol that facilitates the lesioned CST and inhibits the contralesional CRST to improve upper extremity rehabilitation in individuals with moderate-to-severe motor impairments post-stroke.

**Methods:**

Fourteen individuals (minimum 3 months post ischemic stroke) consented. Physician decision of the participants baseline assessment qualified eight to continue in a randomized, double-blind cross-over pilot trial (ClinicalTrials.gov Identifier: NCT05174949) with: (1) anodal high-definition transcranial direct stimulation (HD-tDCS) over the ipsilesional primary motor cortex (M1), (2) cathodal HD-tDCS over contralesional dorsal premotor cortex (PMd), (3) sham stimulation, with a two-week washout period in-between. Subject-specific MR images and computer simulation were used to guide HD-tDCS and verified by Transcranial Magnetic Stimulation (TMS) induced Motor Evoked Potential (MEP). The motor behavior outcome was evaluated by an Fugl-Meyer Upper Extremity score (primary outcome measure) and the excitability of the ipslesoinal CST and contralesional CRST was determined by the change of MEP latencies and amplitude (secondary outcome measures).

**Results:**

The baseline ipsilesional M1 MEP latency and amplitude were correlated with FM-UE. FM-UE scores were improved post HD-tDCS, in comparison to sham stimulation. Both anodal and cathodal HD-tDCS reduced the latency of the ipsilesional M1 MEP. The contralesional PMd MEP disappeared/delayed after HD-tDCS.

**Discussion:**

These results suggest that HD-tDCS could improve the function of the lesioned corticospinal tract and reduce the excitability of the contralesional cortico-reticulospinal tract, thus, improving motor function of the upper extremity in more severely impaired individuals.

## 1. Introduction

Stroke is the leading cause of serious long-term disability in the world. Ischemic strokes, accounting for 87% of all strokes, occur when a vessel supplying blood to the brain is obstructed (Barthels and Das, 2020). 80% of ischemic stroke survivors report movement impairment on the side of the body contralateral to the lesioned hemisphere (Parker et al., 1986). Upper-extremity motor impairments include muscle weakness, abnormal muscle synergies, and spasticity (Parker et al., 1986). Despite the development of many interventions for movement recovery post-stroke, rehabilitation therapies are minimally effective at improving abnormal muscle synergy in more impaired individuals, specifically in the subacute and chronic stages. Late subacute is defined as 3–6 months post stroke and chronic as greater than 6 months (Bernhardt et al., 2017). Both animal and human studies of stroke survivors suggest and support the role of cortico-reticulospinal tract (CRST) hyperexcitability in the contralesional hemisphere in more severe impairments post-stroke (Li, 2017; Li et al., 2019), in particular, the expression of the prevalent abnormal muscle synergies in the paretic upper limb (McPherson et al., 2018a; Tian et al., 2021). CRST hyperexcitability in the contralesional hemisphere emerges as a consequence resulting from damage to the ipsilesional motor cortex or its descending pathway, i.e., the corticospinal tract (CST) (Li et al., 2019). The medial CRST primarily originates from the dorsal premotor cortex (PMd) and travels through the pontine reticular formation (Jang and Lee, 2019). Previous studies applying transcranial magnetic stimulation (TMS) to patients after stroke demonstrated that the medial CRST is responsive to the excitatory ipsilateral input from the PMd in the contralesional hemisphere (Bestmann et al., 2010; Schwerin et al., 2011). This finding makes the contralesional PMd (cPMd) a potential target for combating moderate-to-severe movement impairment.

Recent studies demonstrated that non-invasive neuromodulation technologies, such as *transcranial direct current stimulation* (tDCS) could be a safe and quick approach to modulate cortical excitability (Orrù et al., 2020). Different from other technologies such as robots, functional electrical stimulation, and local vibrations that manipulate the periphery, tDCS modulates brain circuitry directly and facilitates neuroplasticity (Allman et al., 2016). However, the effect of conventional tDCS is limited as it uses large size “sponge” electrodes, making it difficult to target a specific region of interest in the brain for testing the hypothesis. To address the limitation of conventional tDCS that non-specifically activates many brain areas, this study proposes the use of a novel targeted *high-definition tDCS (HD-tDCS)* using a few small electrodes, navigated by subject-specific MR-based computer simulation (Mackenbach et al., 2020) and verified by TMS localization technique, to specifically modulate the targeted cortical regions. The overall objective of this proof-of-concept pilot study is to explore the potential of targeted HD-tDCS to modulate the excitability of specific cortical motor regions and their underlying motor pathways (i.e., CST/CRST) to diminishing post-stroke upper limb impairments, specifically in more impaired individuals. The study is significant because it targets a group of stroke survivors who have limited options for improving upper limb movement ability. In this study, the motor behavior outcome was evaluated by an Fugl-Meyer Upper Extremity score (primary outcome measure), and the excitability of the CST/CRST was determined by the change of MEP latencies and amplitude (secondary outcome measures) (Gladstone et al., 2002). Our key hypotheses are that: (1) facilitating the ipsilesional primary motor cortex (iM1) improves the excitability of the damaged CST, thus, reducing the CRST hyperexcitability and motor impairments, (2) inhibiting the contralesional dorsal premotor cortex (cPMd) directly reduces the CRST hyperexcitability and thus, may also improve motor behaviors.

## 2. Materials and methods

### 2.1. Participants

The study was approved by the internal review board (IRB) of the University of Oklahoma Health Sciences Centre (IRB # 14011). The study was conducted at the University of Oklahoma Health Science Centre, Oklahoma City, OK from January 2022 to June 2023. Fourteen participants with ischemic stroke (at least 3 months post stroke) (four females) consented for the study. One participant (S13) was lost to follow up. The rest of the participants (*n* = 13) were screened at their baseline using the Fugl-Meyer upper extremity (FM-UE) score (Gladstone et al., 2002) and transcranial magnetic stimulation (TMS)-induced motor evoked potentials (MEP). The demographics of participants are provided in Table 1.

**Table 1:** Stroke participants demographics.

Subject ID	Lesion side	Paretic side	Age	Sex	Time post stroke	FM-UE (Total:66)
S1	L	R	64	M	33 months	8
S2*	R	L	72	M	17 months	14
S3*	L	R	81	F	14 months	10
S4	Both	L	55	M	6 months	46
S5*	L	R	44	M	3 months	26
S6	R	L	62	M	30 months	48
S7	L	R	43	M	87 months	53
S8	R	L	59	M	33 months	46
S9*	R	L	65	M	14 months	16
S10*	L	R	73	F	92 months	23
S11*	R	L	57	F	7 months	15
S12*	L	R	67	M	11 months	16
S13	R	L	75	F	5 months	-
S14*	R	L	38	M	4 months	38

^*^Participants who attended HD-tDCS sessions.

### 2.2. Baseline procedure

**Figure 1:**
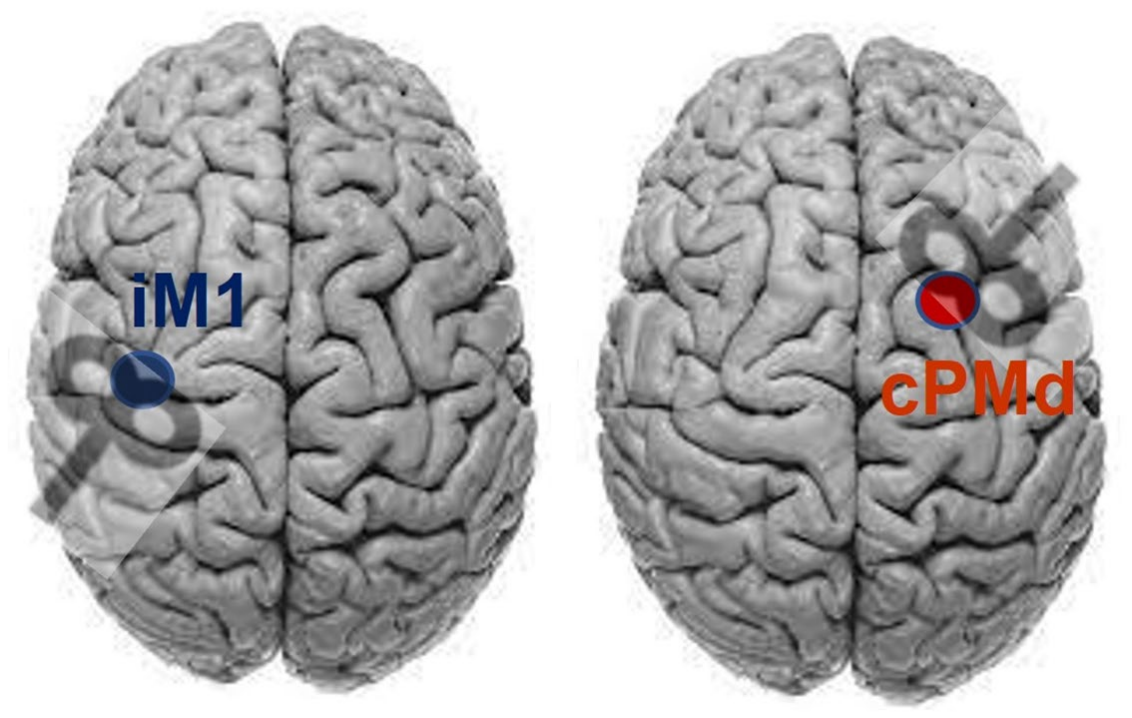
Coil orientation for stimulating ipsilesional primary motor cortex (iM1) and contralesional dorsal premotor area (cPMd), assuming the lesion on the left side.

The FM-UE was performed by a licensed physical therapist. The TMS-induced MEP were assessed to determine the use of the ipsilesional corticospinal tract and the contralesional cortico-reticulospinal tract (Bestmann et al., 2010; Schwerin et al., 2011), with the MEP latency/status as the outcome measure (Schwerin et al., 2008, 2011). The paired-pulse TMS (Magstim® BiStim2, The Magstim Company Ltd., Spring Gardens, Whitland, United Kingdom) was applied at the respective hotspots for the elbow flexor muscle at the paretic arm, i.e., Biceps Brachii, over the ipsilesional primary motor cortex (from which the corticospinal tract originates) and contralesional dorsal premotor cortex (from which the cortico-reticulospinal tract originates) with reference to the paretic arm, using a figure-eight coil (Schwerin et al., 2011). A paired-pulse TMS with a conditioning pulse (65% stimulator maximum intensity) followed by a testing pulse (85% stimulator maximum intensity) was used to avoid the need to pre-activate the muscle (which could cause the bias of background EMG) (Oliveri et al., 2000), with paired-pulse intervals of 25 ms (Schwerin et al., 2011). The center of the coil was positioned tangentially to the skull with the handle at 45° from the parasagittal plane: posterior–anterior orientation for ipsilesional M1 and anterior–posterior orientation for contralesional PMd (Figure 1) (Tscherpel et al., 2020a,b). The M1 hotspot is defined as the grid-point that results in the largest response in the target muscle, and was found for the ipsilesional M1 and contralesional M1 hemisphere through stimulation of a 5 × 5 grid of 1 cm spaced sites on the scalp over motor areas of each hemisphere (centered at C3/4 of 10–20 EEG system) (Schwerin et al., 2008). The “hot-spot” of the contralesional PMd was identified using a reference point of 1 cm medial and 2.5 cm anterior of the M1 “hot-spot” at the contralesional hemisphere (Stephan et al., 2016; Tscherpel et al., 2020b). The MEP status was determined using criteria previously reported (Stinear et al., 2017): the patient was considered MEP+ if MEPs of any amplitude are observed at a consistent latency on at least 5 out 10 trials; otherwise, MEP–. After determining the status of MEP, at least eight more pulses (inter-stimulus interval: 2–3 s) were applied to the identified hotspot to get a robust estimate of the latency of the MEP. Together with determination trials, the average latency was calculated across all positive trials (more than 18 trials of MEP+) to determine the latency and amplitude of MEP.

### 2.3. HD-tDCS procedure

After the baseline assessment, eight of the participants (S2, S3, S5, S9, S10, S11, S12, and S14) who were more severely impaired (FM-UE: 10–38), had a detectible ipsilateral MEP from the contralesional PMd, and met the inclusion/exclusion criteria of a registered pilot clinical trial “Targeted High-definition Transcranial Direct Current Stimulation (HD-tDCS) for Reducing Post-stroke Movement Impairments” (ClinicalTrials.gov Identifier: NCT05174949) were included in this pilot trial.

#### 2.3.1. Inclusion criteria

- Ischemic unilateral, subcortical stroke lesion (confirmed by the most recent clinical or radiological reports) at least 3 months prior to participation in this project.
- Paresis confined to one side, with moderate to severe motor impairment of the upper limb (Fugl-Meyer upper extremity scores between 10 and 40 out of 66 at the first visit of this study).
- Capacity to provide informed consent.

#### 2.3.2. Exclusion criteria

- Muscle tone abnormalities and motor or sensory impairment in the unimpaired limb.
- Severe wasting (Fugl-Meyer upper extremity scores below 10) or contracture or significant sensory deficits in the paretic upper limb.
- Severe cognitive or affective dysfunction that prevents normal communication and understanding of consent or instruction.
- Severe concurrent medical problems (e.g., cardiorespiratory impairment).
- Using a pacemaker.
- Metal implants in the head.
- Known adverse reaction to TMS and tDCS.
- Pregnant.

These eight participants continued to participate in this randomized, double-blind (Participant, Outcomes Assessor) cross-over pilot trial with three visits: (1) anodal high-definition transcranial direct stimulation (HD-tDCS) over the ipsilesional M1, (2) cathodal HD-tDCS over contralesional PMd, (3) sham stimulation, with a two-week washout period in-between. The order in which the participants received the stimulations (anodal, cathodal, and sham) was computer randomized.

**Figure 2:**
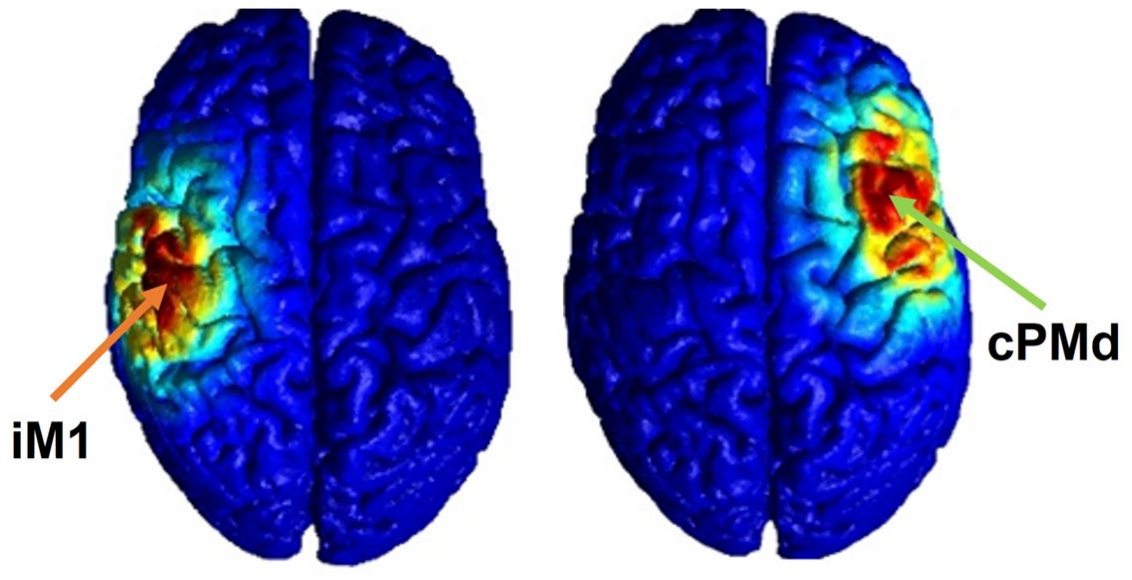
Electrical field estimation for ipsilesional M1 (left) and contralesional PMd (right) HD-tDCS.

The proposed HD-tDCS method uses five small (1 centimeter in diameter) electrodes with the main stimulation electrode in the center, and four surrounding co-centric electrodes with opposite polarity. The HD-tDCS electrodes (4×1 HD-tDCS unit, Soterix Medical Inc., Woodbridge, New Jersey, United States) were mounted onto a standard 10–20 EEG cap. The stimulation dosage was set as 2 mA, for 20 min, the optimal safe dosage to influence neuroplasticity according to the safety guidelines of HD-tDCS (Gbadeyan et al., 2016; Godinho et al., 2017). For sham stimulation, the HD-tDCS unit was set to the automatic sham feature, which produces a sham waveform based on the indicated “real” waveform by only ramping the current to 2 mA at the start and end of the stimulation to provide the same feeling as active stimulation to the participants. The stimulation location was identified using subject-specific 1.5 T MR images (the T1 weighted images were obtained by using a T1 SAG FLAIR sequence with FOV = 22 cm, Slice Thickness: 5 mm and the T2 weighted images were obtained by suing T2 AX sequence with the same FOV and Slide Thickness values as the T1) and verified by the TMS-induced MEP as explained, with the center electrode on the TMS “hot-spot” and 40–45 mm (depending on the size of the head) distance between the center and surrounding electrodes (Schwerin et al., 2008, 2011). This is the optimal distance based on our previous simulation study (Mackenbach et al., 2020). Electrical fields in the brain were estimated using the Realistic Volumetric Approach to Simulate Transcranial Electric Stimulation (ROAST) toolbox to confirm that the targeted brain area was stimulated (as illustrated in Figure 2) (Huang et al., 2019). To run these simulations, the participants T1 and T2 weighted MR images were inputted into the pipeline and the default electrical conductivities were used: white matter (0.126 S/m); gray matter (0.276 S/m); CSF (1.65 S/m); bone (0.01 S/m); skin (0.465 S/m); air (2.5e-14 S/m); gel (0.3 S/m); electrode (5.9e7 S/m) (Huang et al., 2019). In the simulation, a central electrode was placed over the target area with four surrounding electrodes placed in a circle at equal distance with opposite polarity (Mackenbach et al., 2020).

### 2.4. Statistical analysis

The effect of HD-tDCS is determined by the change in FM-UE score (primary outcome measure), and the change of MEP latencies and amplitude (secondary outcome measures). All statistical analysis was completed using commercial software Statistical Analysis Systems (9.4, SAS, Carey, NC, United States). After checking for and finding no evidence of a non-normal outcome measure distribution, the data was analyzed using generalized estimating equation (GEE) using PROC GENMOD. This method was selected due to its ability to improve the power in small-sample studies in which the temporal spacing of outcomes is the same for each subject. Specifically, we use a modified empirical sandwich covariance matrix estimator within correlation structure selection criteria and test statistics. Use of this estimator can improve the accuracy of selection criteria and increase the degrees of freedom to be used for inference (Westgate and Burchett, 2016). The fixed factors are group (anodal, cathodal, sham), time (pre and post intervention), with their interaction, and the random factor is subject ID. This technique uses correlated linear models for each outcome variable.

## 3. Results

**Figure 3:**
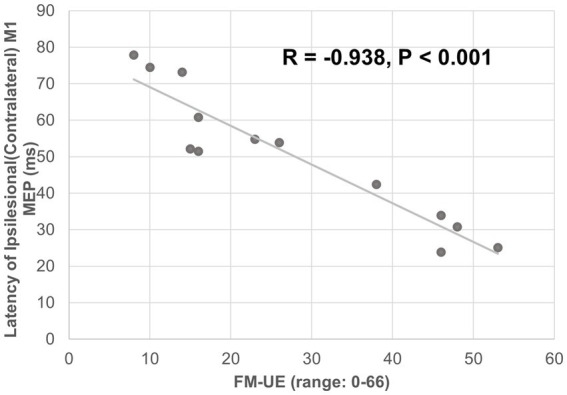
Correlation between the latency of ipsilesional (contralateral) M1 MEP and FM-UE.

**Figure 4:**
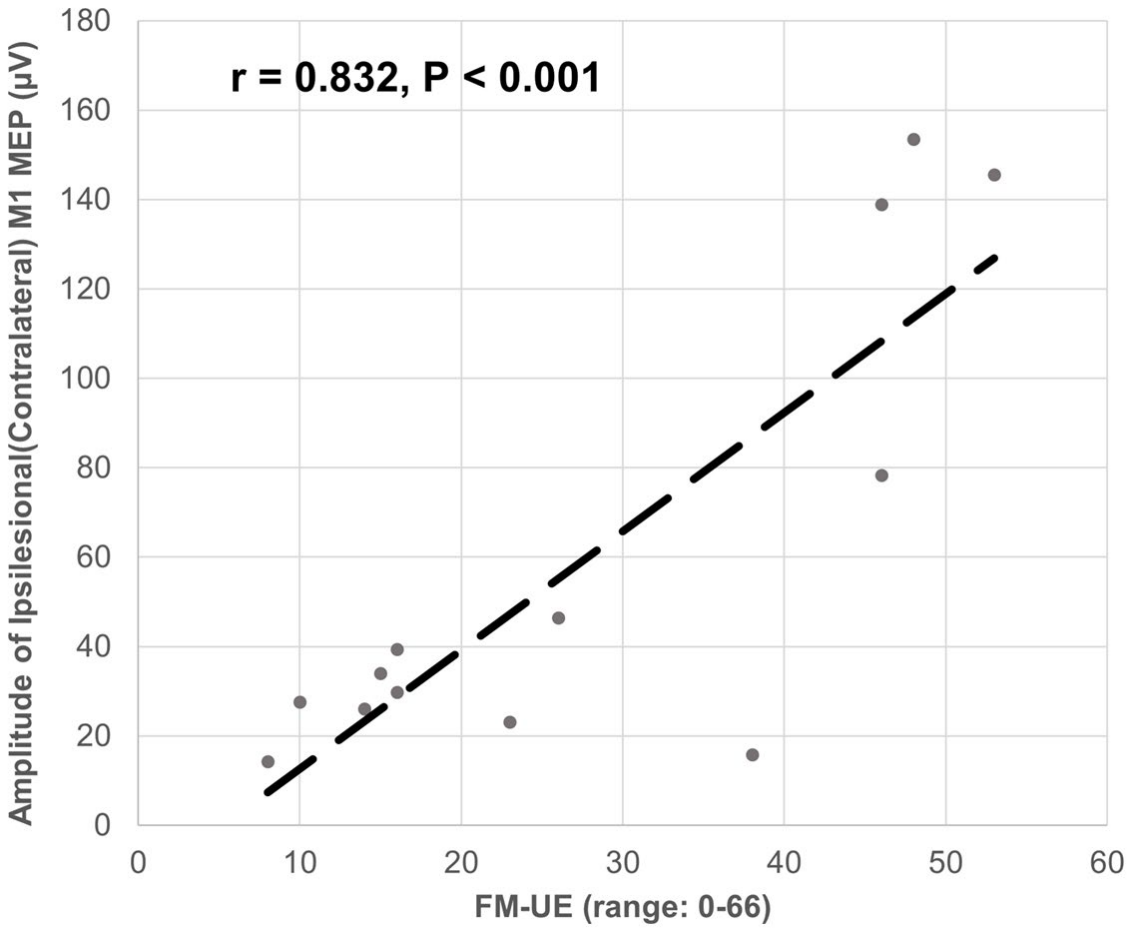
Correlation between the amplitude of ipsilesional (contralateral) M1 MEP and FM-UE.

The ipsilesional M1-induced MEP of the impaired arm was detected at the baseline for all 13 participants. The latency of the ipsilesional M1-induced MEP was negatively correlated with FM-UE score (correlation coefficient *r* = − 0.938, *p* < 0.001). Moreover, the latency of the ipsilesional M1 MEP was predictive of FM-UE scores (determination of correlation: *R*^2^ = 0.880) (Figure 3). The amplitude of the ipsilesional M1-induced MEP was positively correlated with FM-UE score (correlation coefficient *r* = 0.832, *p* < 0.001). The amplitude of ipsilesional M1 MEP was also predictive of FM-UE scores (determination of correlation: *R*^2^ = 0.692) (Figure 4).

**Figure 5:**
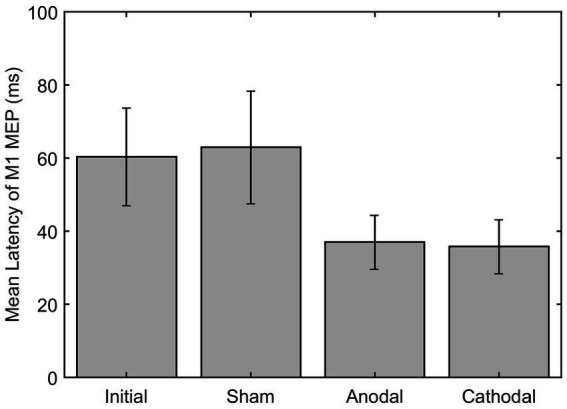
Latency of M1 MEP on ipsilesional M1 pre and post HD-tDCS stimulation (Sham, Anodal and Cathodal).

For the eight moderate-to-severe subjects (S2, S3, S5, S9, S10, S11, S12, and S14) who qualified for the study and attended the HD-tDCS sessions, GEE analysis of the ipsilesional M1 MEP latency revealed that the anode mean difference (−24.21 ms) was significantly different from the mean difference of the sham (1.16 ms) *p* = 0.0020. Additionally, when compared to the sham, the anode group (group*time) changed significantly differently after intervention with a beta estimate of-25.37, *z* = −9.03, and *p* < 0.0001. Similarly, the cathode mean difference (−22.2) was also significantly different from the sham *p* = 0.0088. In addition, the cathode group (group*time) also changed significantly differently over time compared to the sham with beta estimate of −23.36, *z* = −8.034, and *p* < 0.0001 (see Figure 5). There was no significant statistical difference or interaction between either anodal or cathodal stimulation compared to sham on MEP amplitude.

As a comparison, the contralesional M1-induced MEP of the non-impaired arm was also measured for reference (14.77 ± 1.52 ms) which is in line with previously reported range of M1-induced MEP for non-impaired arm in the literature (Müller et al., 1997; Jacques et al., 2023). The contralesional M1 MEP did not change after the HD-tDCS (14.49 ± 1.43 *p* > 0.05). Only moderate-to-severe participants had a detectible ipsilateral MEP response on the impaired arm when stimulating the contralesional PMd. The contralesional PMd MEP either disappeared or was delayed after active (anodal/cathodal) HD-tDCS but not after the sham stimulation (Table 2).

**Table 2:** Latency of contralesional PMd MEP.

Subject ID	Initial	Sham	Anodal	Cathodal
S2	58.47 ms	63.65 ms	(−)	(−)
S3	89.96 ms	90.88 ms	107.05 ms	106.73 ms
S5	91.03 ms	88.60 ms	(−)	103.53 ms
S9	86.34 ms	92.70 ms	105.48 ms	(−)
S10	82.31 ms	76.60 ms	110.76 ms	(−)
S11	88.76 ms	79.61 ms	(−)	142.06 ms
S12	61.64 ms	54.61 ms	(−)	(−)
S14	63.67 ms	66.97 ms	(−)	96.85 ms

**Figure 6:**
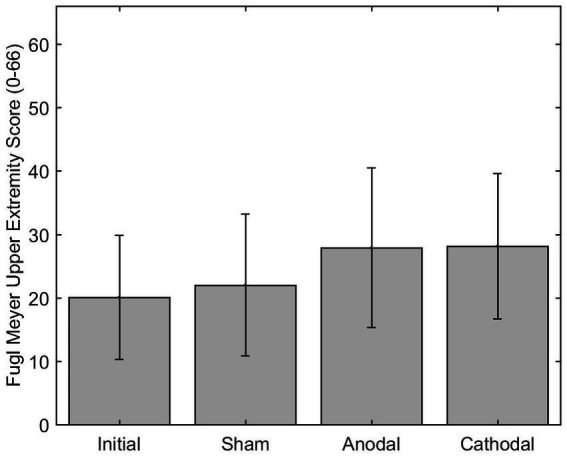
FM-UE score pre and post HD-tDCS stimulation (Sham, Anodal, and Cathodal).

The mean difference of the FM-UE (scored 0–66) after anodal stimulation (7.88) was significantly difference from the sham mean difference (2.00) *p* = 0.0344. Additionally, the change in FM-UE over time post anodal HD-tDCS (group*time) changed significantly differently with a beta estimate of 5.875 with *z* = 2.72 and *p* = 0.0066, when compared to the sham group. The cathode had a mean difference of 8.13 for the FM-UE, which was significantly different than sham *p* < 0.0001. Additionally, the cathode group (group*time) also changed significantly differently over time compared to the sham with beta estimate of 5.125, *z* = 4.04, and *p* < 0.0001 (Figure 6). The minimally clinically significant difference for the FM-UE is 5 points.

## 4. Discussion

In this study of HD-tDCS, we utilized a cross-over study design in which each person was able to serve as his or her own control. This increased the power of this study three-fold and allowed us to achieve significant results with the eight patients who completed the trial. These patients were in various stages of motor recovery which further strengthens the results of this study, as it demonstrates the technique is useful within all stages of time post stroke.

This study provides preliminary support of the use of both targeted anodal and cathodal HD-tDCS as a method for stroke rehabilitation in those with severe motor impairments. The relationship between the FM-UE motor score and the latency and amplitude of contralateral ipsilesional M1 TMS-induced MEP is consistent with prior studies on MEPs and clinical assessments post-stroke (Escudero et al., 1998; Karatzetzou et al., 2022; Li et al., 2022). This finding confirmed that change in MEPs have potential to be good predictors of functional change of motor descending pathways post HD-tDCS stimulation and the selection of them outcome measures of this study.

We observed that facilitating ipsilesional M1 using anodal HD-tDCS decreased the latency of ipsilesional M1 TMS-induced MEP, as the change in latency was significantly different between anodal and sham stimulation. The anodal stimulation also improved FM-UE score by an average of 7.88 points, as compared to the sham 2 points on average. The minimally clinically important difference for the FM-UE ranges from 4.25–7.25 points (Hiragami et al., 2019). The increase in FM-UE scores reflects an improvement in overall motor impairment of the upper extremity post anodal stimulation. This improvement is consistent with prior studies on anodal HD-tDCS at ipsilesional M1 post stroke (Bao et al., 2019; Kim et al., 2022). However, this study further demonstrates that anodal stimulation may specifically improve the excitability of the damaged CST and improve motor impairments. More importantly, the facilitation of the CST may also reduce hyperexcitability in the CRST, as post anodal stimulation the latency of contralesional PMd TMS-induced MEP was either delayed, or not detected. This is likely because the increased cortical excitability of ipsilesional M1 may enhance the super bulbar inhibition to the reticulospinal tract via the cortico-reticular pathways (Jang and Lee, 2019). While the amplitude was correlated to the baseline of impairment with the FM-UE score, there was not a significant change in amplitude pre and post HD-tDCS stimulation. The results of the change in MEP amplitude post tDCS stimulation are highly variable in the literature (Priori et al., 1998; Samani et al., 2019; Karatzetzou et al., 2022). In fact, recent reviews and studies have shown that MEP amplitude is not a sufficiently robust measure of tDCS at low intensities (Horvath et al., 2015; Pillen et al., 2022). The results of this pilot study provide some preliminary evidence that MEP latency may be a predictor of the neurophysiology effect of tDCS than MEP amplitude.

Furthermore, inhibiting the contralesional PMd decreased the latency of ipsilesional M1 TMS-induced MEP as the change in latency was significantly different between cathodal and sham stimulation. The cathodal stimulation also improved the FM-UE score with an 8.13-point increase on average. Noteworthy, the targeted HD-tDCS used in this study precisely modulated the excitability of contralesional PMd without significantly changing contralesional M1 excitability (no significant changes to contralesional M1 MEP). This indicates that inhibiting the contralesional PMd leads to a reduced recruitment of CRST, which is known as the key drive of post-stroke spasticity (Li et al., 2019). Based on previous research, this result may be related to decreased input to descending monoaminergic pathways at the ponto-medullary reticular formation that reduce the hyperactivity of alpha motoneuron pool at spinal cord (McPherson et al., 2018b).

This finding may play a role in developing an alternative intervention for severely impaired stroke survivors exhibiting increased levels of spasticity. Despite the development of a variety of interventions for movement recovery, rehabilitation treatments are minimally effective for more impaired individuals, making this finding especially clinically relevant. Currently, botulinum toxin has been increasingly used to treat upper limb spasticity post-stroke. Botulinum toxin is injected locally into the muscle and causes temporary paresis by blocking cholinergic transmission (Dolly and Aoki, 2006). While this can reduce muscle tone, there is currently not a significant difference in improved arm function (Shaw et al., 2011; Teasell et al., 2012; Santamato et al., 2013).

Overall, this study improves our understanding of neural circuitry and plasticity post stroke by confirming neural targets (ipsilesional M1 and contralesional PMd) for motor descending pathways. It also shows the benefit of subject specific precise neuro-navigation to guide the stimulation. The promising result for more impaired individuals is highly significant as it may provide an alternative intervention option to those with limited options for improving their upper limb function. However, due to the sample size, a future large-scale study is required to translate this research to clinical practice.

### 4.1. Limitations and future work

This pilot trial involved a few stroke subjects who are at least 3 months post stroke. The aim was to exclusively examine the feasibility of HD-tDCS on modulating the function of motor descending pathways. In the first 3 months, stroke survivors typically experience some degree of spontaneous motor and sensory recovery (Cramer, 2008) and may attend a physical therapy rehabilitation program (Dromerick et al., 2021). These co-variants would make it difficult to determine the sole impact of HD-tDCS. After the first 3 months in stroke recovery, most spontaneous motor and sensory improvement reaches a plateau (Grefkes and Fink, 2020). Therefore, this study used participants at least 3 months post stroke with a cross-over study design to reduce type I error. However, this protocol could be a useful addition to the acute phase. Additionally, having stroke subjects in various stages of subacute and chronic could have been a reason for the variation in results. Hence, future work would be a larger clinical trial involving acute, subacute, and chronic stroke participants in a parallel group trial. Furthermore, future study could include the addition of physical exercise to further explore its potential to improve current intervention practice of stroke rehabilitation.

## Data availability statement

The raw data supporting the conclusions of this article will be made available by the authors, without undue reservation.

## Ethics statement

The studies involving humans were approved by the study was approved by the internal review board (IRB) of the University of Oklahoma Health Sciences Centre (IRB # 14011). The studies were conducted in accordance with the local legislation and institutional requirements. The participants provided their written informed consent to participate in this study.

## Author contributions

JW: Data curation, Formal analysis, Writing-original draft. SJ: Data curation, Formal analysis, Supervision, Validation, Writing – review & editing. DH: Data curation, Writing – review & editing. SL: Writing – review & editing. ES: Resources, Writing – review & editing. YY: Conceptualization, Funding acquisition, Investigation, Project administration, Supervision, Writing – original draft, Writing – review & editing.
