# Corrigendum: High-definition transcranial direct current stimulation for upper extremity rehabilitation in moderate-to-severe ischemic stroke: a pilot study

**DOI:** 10.3389/fnhum.2024.1449239

**Published:** 2024-06-27

**Authors:** Jordan N. Williamson, Shirley A. James, Dorothy He, Sheng Li, Evgeny V. Sidorov, Yuan Yang

**Affiliations:** ^1^Department of Bioengineering, Grainger College of Engineering, University of Illinois Urbana-Champaign, Urbana, IL, United States; ^2^University of Oklahoma Health Sciences Center, Hudson College of Public Health, Oklahoma City, OK, United States; ^3^University of Oklahoma Health Sciences Center, College of Medicine, Oklahoma City, OK, United States; ^4^Department of Physical Medicine and Rehabilitation, UT Health Huston, McGovern Medical School, Houston, TX, United States; ^5^Department of Neurology, University of Oklahoma Health Sciences Center, Oklahoma City, OK, United States; ^6^Clinical Imaging Research Center, Stephenson Family Clinical Research Institute, Carle Foundation Hospital, Urbana, IL, United States; ^7^Beckman Institute for Advanced Science and Technology, University of Illinois Urbana-Champaign, Urbana, IL, United States; ^8^Department of Physical Therapy and Human Movement Sciences, Northwestern University, Chicago, IL, United States; ^9^Department of Rehabilitation Sciences, College of Allied Health, University of Oklahoma Health Sciences Center, Oklahoma City, OK, United States; ^10^Gallogly College of Engineering, Stephenson School of Biomedical Engineering, University of Oklahoma, Oklahoma City, OK, United States

**Keywords:** transcranial direct current stimulation, transcranial magnetic stimulation, stroke, upper extremity rehabilitation, motor evoked potential

In the published article, there was an error in the Funding statement. The funding sources NIH R01HD109157 and the National Science Foundation (NSF 2236459) did not support the research, authorship, and/or publication of this paper, and have since been removed. The corrected Funding statement appears below.

